# Ultraviolet Photodetectors Based on 4H‐SiC With Honeycomb‐Like Light‐Trapping Structures

**DOI:** 10.1002/advs.74352

**Published:** 2026-02-11

**Authors:** Huifan Xiong, Xiliang Luo, Qunsi Yang, Yibo Hu, Jieshi Chen, Lihui Song, Deren Yang, Xiaodong Pi

**Affiliations:** ^1^ State Key Laboratory of Silicon and Advanced Semiconductor Materials & School of Materials Science and Engineering Zhejiang University Hangzhou China; ^2^ Key Laboratory of Power Semiconductor Materials and Devices of Zhejiang Province & Institute of Advanced Semiconductors ZJU‐Hangzhou Global Scientific and Technological Innovation Center Zhejiang University Hangzhou China; ^3^ School of Materials Science and Engineering Shanghai University of Engineering Science Shanghai China

**Keywords:** light‐trapping, photoelectrochemical etching, self‐power, silicon carbide

## Abstract

Silicon carbide (SiC), as a wide‐bandgap semiconductor, exhibits significant potential for visual‐blind ultraviolet (UV) detection due to its excellent material properties. To further improve the performance of SiC UV detectors, we introduce the honeycomb‐like light‐trapping microstructures at the surface of SiC, which are fabricated by using a facile and efficient photoelectrochemical (PEC) etching method. The mechanism underlying the formation of the light‐trapping microstructures is carefully investigated. It is found that the anisotropic etching is dependent on the crystal orientation of SiC. After the formation of the honeycomb‐like light‐trapping microstructures, optical characterizations reveal substantial suppression of UV reflection and enhanced absorption due to the increased light path and multiple internal reflections. The self‐powered SiC UV photodetector with the light‐trapping microstructures shows a peak responsivity of 0.187 A/W at the wavelength of 290 nm, which corresponds to an external quantum efficiency of 80%. This self‐powered photodetector also demonstrates excellent performance in imaging and optical communication. The low‐cost, scalable, and selective etching for the formation of the light‐trapping microstructures has important implications for the development of high‐performance SiC‐based optoelectronic devices.

## Graphical abstract

A facile photoelectrochemical etching method creates honeycomb‐like light‐trapping microstructures on SiC. These microstructures suppress UV reflection and enhance absorption by extending the optical path and facilitating multiple internal reflections, while strengthening the local electric field. The resulting self‐powered SiC UV photodetector achieves a responsivity of 0.187 A/W and an EQE of 80% at 290 nm, demonstrating excellent potential for imaging and communication.

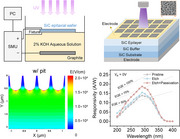

## 1 Introduction

Silicon carbide (SiC) is a wide‐bandgap semiconductor known for its outstanding electrical, optical, and thermal properties. It has become a fundamental material for next‐generation power electronics and radiation‐hardened systems [1, 2, 3, 4, 5, 6]. Its bandgap of ∼3.2 eV corresponds to a cut‐off wavelength around 390 nm, which makes it highly suitable for visual‐blind ultraviolet (UV) detection [7, 8]. UV photodetectors are increasingly important in numerous fields, including environmental monitoring, flame detection, space exploration, and secure communications [9, 10, 11, 12]. SiC‐based UV detectors are particularly attractive due to their compact size, high sensitivity, excellent visible‐blindness, low power consumption, and high reliability [13, 14, 15, 16].

A major challenge limiting the performance of SiC UV detectors is the high refractive index of SiC [4, 17, 18], which leads to significant Fresnel reflection losses at the air‐SiC interface. This reduces the amount of light entering the active region and thus constrains the overall efficiency of UV detectors. To address this issue, dry etching techniques have been widely used to fabricate light‐trapping structures, such as nanorods and nano‐cone holes, on SiC surfaces [19, 20]. Although dry etching allows precise morphological control, it suffers from high equipment cost, limited throughput, and potential surface damage caused by ion bombardment [21, 22, 23]. Wet chemical etching offers a more cost‐effective alternative for surface texturing, as demonstrated by its successful application in forming pyramidal light‐trapping structures in silicon solar cells [24, 25, 26]. Nevertheless, conventional wet etching for SiC material is very slow with common alkaline solutions due to the significant chemical stability of SiC material. Photoelectrochemical (PEC) etching has emerged as an alternative method, which can dramatically enhance the etching rate of SiC material and is, moreover, effective and cost‐efficient [27, 28, 29]. But up to date, most studies of PEC etching on SiC have focused on etching on the highly doped substrates [27, 28, 29, 30, 31], which are typically not the light‐incident side in photodetector configurations. In contrast, etching on the lower‐doped epilayers, which serve as the active region for photon absorption and carrier transport, has received less attention. Furthermore, the behaviors of PEC etching on SiC epilayers remain nearly not studied. The challenge lies in the fact that the C‐face of the SiC substrate exhibits a faster chemical reaction rate compared to the Si‐face of the SiC epilayer [32, 33]; it is hard to achieve selective etching only on the SiC epilayer.

In this work, we develop a PEC etching method using a KOH aqueous solution to fabricate light‐trapping microstructures selectively on the SiC epilayer surface. To optimize the light trapping microstructures, we design a simple etching process that preserves the substrate while enabling uniform epilayer texturing. This texturing results in the honeycomb‐like microstructures, and then the formation mechanisms underlying it are systematically investigated. The etched samples demonstrate significantly reduced UV reflection and enhanced UV absorption. Last, we fabricate a self‐powered SiC UV photodetector (PD) incorporating the honeycomb‐like light‐trapping microstructures, demonstrating markedly improved responsivity of 0.187 A/W at 290 nm. Meanwhile, this device demonstrates potential for imaging and optical communication applications. Overall, this work provides a scalable and low‐cost approach to enhancing the performance of the SiC‐based UV detectors.

## 2 Experimental Section

### 2.1 Device Fabrication

The SiC epitaxial wafer used in this work was grown homoepitaxially on a 6‐inch production‐grade SiC substrate via chemical vapor deposition (CVD). The material exhibits excellent crystal quality, with supporting evidence from various characterization results presented in Figure S1. The epilayer has a thickness of 30 µm and a doping concentration of ∼2.9×10^14^ cm^−^^3^. The substrate was 350 µm thick with a resistivity of 20 mΩ·cm. A 1 um thick buffer layer with a doping level of 1×10^18^ cm^−3^ was also incorporated between the substrate and epilayer. The wafer was diced into 1.5 cm ×1.5 cm pieces. Then these samples underwent standard RCA cleaning to ensure surface purity.

Ohmic contact was formed on the substrate side by sputtering a Ni grid hard mask followed by rapid thermal annealing (RTP) at 1000°C for 1 min. On the epilayer side, grid Schottky contacts were fabricated using photolithography and Ni sputtering. Subsequently, PEC etching was applied to form light‐trapping microstructures on the epilayer, after which the epilayer surface was passivated with a 20 nm thick Al_2_O_3_ layer deposited via atomic layer deposition (ALD).

### 2.2 Photoelectrochemical Etching

PEC etching was performed in a two‐electrode electrochemical cell with a graphite cathode. The SiC sample was mounted on the anode such that only the epilayer surface was in coplanar contact with the etching solution, a 2% aqueous KOH solution, while the substrate faced a platinum sheet and remained isolated from the electrolyte. Besides, UV illumination during PEC etching was provided by a mercury lamp. The concentration of KOH and the illumination intensity of the mercury lamp used for etching both have significant impacts on the device performance. The comparative results under different etching conditions can be found in Figure S2.

### 2.3 Materials and Device Characterization

Surface morphology was examined using differential interference contrast (DIC) microscopy (Olympus BX53M) and scanning electron microscope (Thermo Fisher Scientific Scios2 Hivac). Raman measurements were performed on the 4H‐SiC materials at room temperature with a Horiba LabRAM Odyssey Raman microscope. X‐ray Diffraction (XRD) analysis was performed on the X'Pert 3 MRD system of Malvern Panalytical. The high‐resolution transmission electron microscope (HRTEM) images were characterized using the F200X S/TEM of Thermo Fisher Talos. The height distribution was measured using a BRUKER ContourX‐200 white light interferometer. Micro‐Raman and micro‐photoluminescence (micro‐PL) spectra were obtained at room temperature using a Horiba LabRAM Odyssey system. Optical reflectance and transmittance were measured using an Agilent Cary 5000 UV‐Vis‐NIR spectrophotometer. Three‐dimensional (3D) surface topography was analyzed via white light interferometry (Bruker CONTOURX‐200).

### 2.4 Photodetectors Performance Measurement

The optoelectronic characteristics of the photodetectors were characterized using a Keithley 2636B source meter unit (SMU). Device response time was tested using a Tektronix TBS 2000B series digital oscilloscope. Please note that monochromatic UV light was generated by an ultraviolet‐enhanced xenon lamp coupled to a monochromator. The UV light intensity at different wavelengths during spectral measurement and the corresponding EQE testing is shown in Figure S6. For imaging and optical communication tests, a 261 nm semiconductor laser was used.

### 2.5 Device Simulation

Two‐dimensional (2D) simulations of the SiC UV photodetector were carried out using the Sentaurus TCAD tool. The model incorporated the actual material parameters and surface morphology data of the light‐trapping microstructures obtained from white light interferometry. The device was simulated as a Schottky junction photodetector, whose barrier height was obtained from *I‐V* curve fitting, as shown in Figure S7.

## 3 Results and Discussion

**FIGURE 1:**
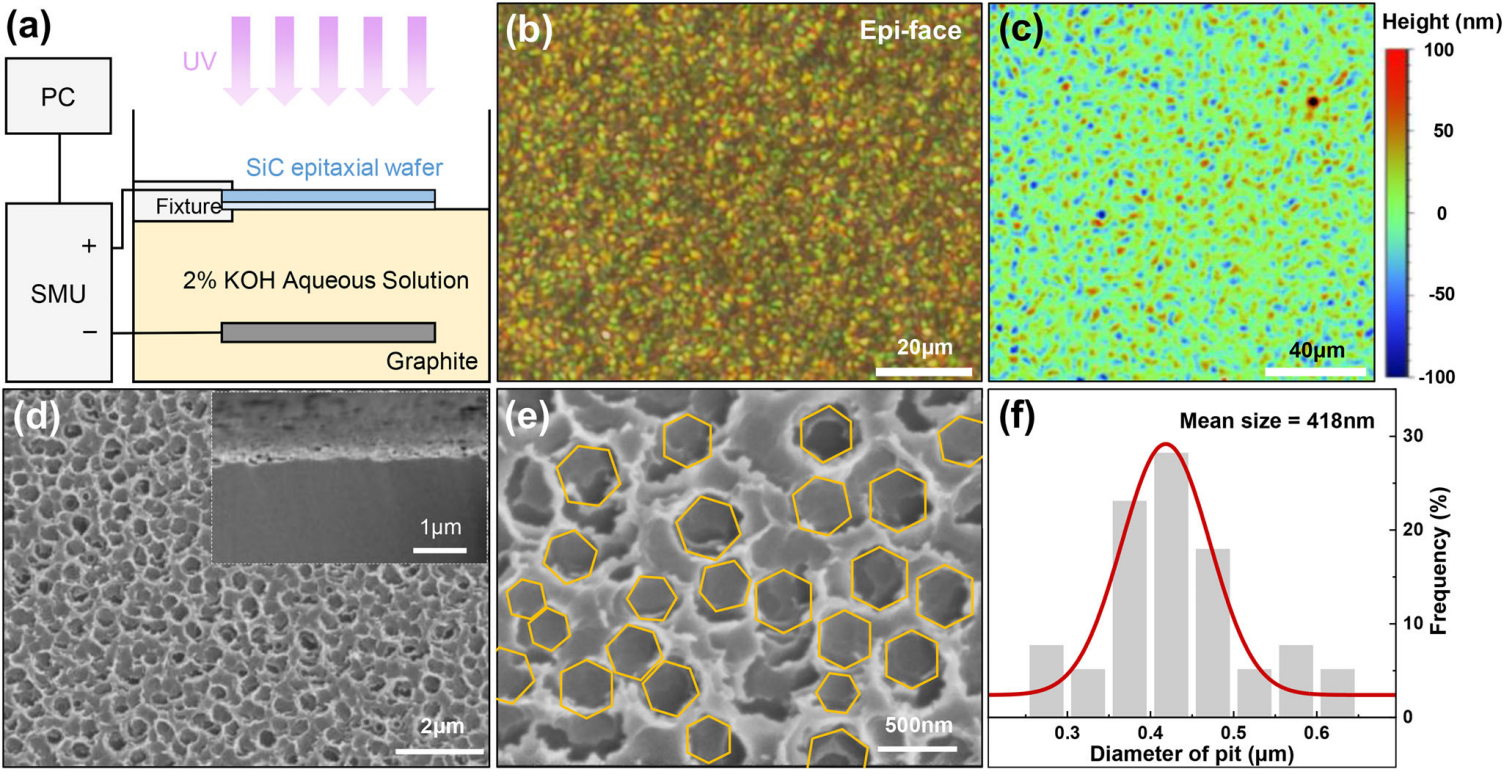
PEC etching of SiC epitaxial wafer and the subsequent morphology characterizations. (a) Schematic diagram of experimental set‐up. The surface of the epitaxial layer (light blue region) of the SiC is in contact with the etching solution surface, whereas the substrate (dark blue region) is facing ultraviolet light. (b) DIC bright field images and (c) height profile measured by white light interferometer of etched epilayer surface. (d) SEM images of surface and cross‐section (insert) of the honeycomb‐like light‐trapping microstructures in SiC epilayer surface after PEC etching at 15 mA current for 2 h, and (e) the enlarged view. (f) Statistical distribution of honeycomb‐like pit dimensions formed by this PEC etching.

The active region of the SiC UV photodetectors is the lower‐doped epilayer. To achieve selective etching of the epilayer surface, a custom fixture was used to position the sample such that the etching solution made coplanar contact only with the epilayer surface, leaving the substrate unaffected (Figure 1a). This approach successfully restricted etching only to the epilayer. A constant‐current PEC etching was conducted for the SiC epitaxial wafer with 15 mA current for 2 h. Differential interference contrast (DIC) microscopy revealed a uniformly rough morphology on the etched epilayer surface (Figure 1b), contrasting with the smooth substrate surface (Figures S3a). The height distribution of the etched sample surface was also tested using a white light interferometer, as shown in Figure 1c, with height variations within 200 nm. Furthermore, a control experiment where the sample was fully submerged in the etching solution resulted in the etching of its substrate surface (Figure S3c). This demonstrated the superiority of the selective etching method. The mechanism of PEC etching on SiC can be explained through energy band diagrams (Figure S4). Upon contact with the KOH solution, band bending occurs at the SiC/solution interface, forming a Schottky barrier that depletes electrons and slightly accumulates holes at the SiC/solution interface. Subsequently, under UV illumination, electron‐hole pairs are generated and then separated by the built‐in electric field. As a result, holes accumulate more and more at the SiC epilayer surface, where they act as oxidizing agents that facilitate the following etching reaction [27, 34, 35], represented by:

(1) $$\mathrm{SiC}+10OH^{-}+8h^{+}\;\to \;\mathrm{Si}{O_{3}}^{2-}+5H_{2}O+CO_{2}$$

The resultant morphology and a statistical analysis of the etched features are further illustrated. Figure 1d provides a scanning electron micrograph (SEM) image of the SiC epilayer surface after PEC etching at 15 mA current for 2 h, revealing a dense array of honeycomb‐like light‐trapping pits. The inset cross‐sectional view indicates that the etching occurred only on the surface of the SiC. Figure 1e offers a higher magnification SEM image of the same etched surface, with the honeycomb‐like pits highlighted by yellow outlines. The presence of hexagonal pits indicates a certain degree of crystallographic selectivity during the PEC etching process, which corresponds with the hexagonal symmetry of the SiC crystal. The histogram in Figure 1f presents the statistical distribution of the honeycomb‐like pit diameters observed on the etched SiC sample surface, revealing an average pit diameter of approximately 418 nm.

**FIGURE 2:**
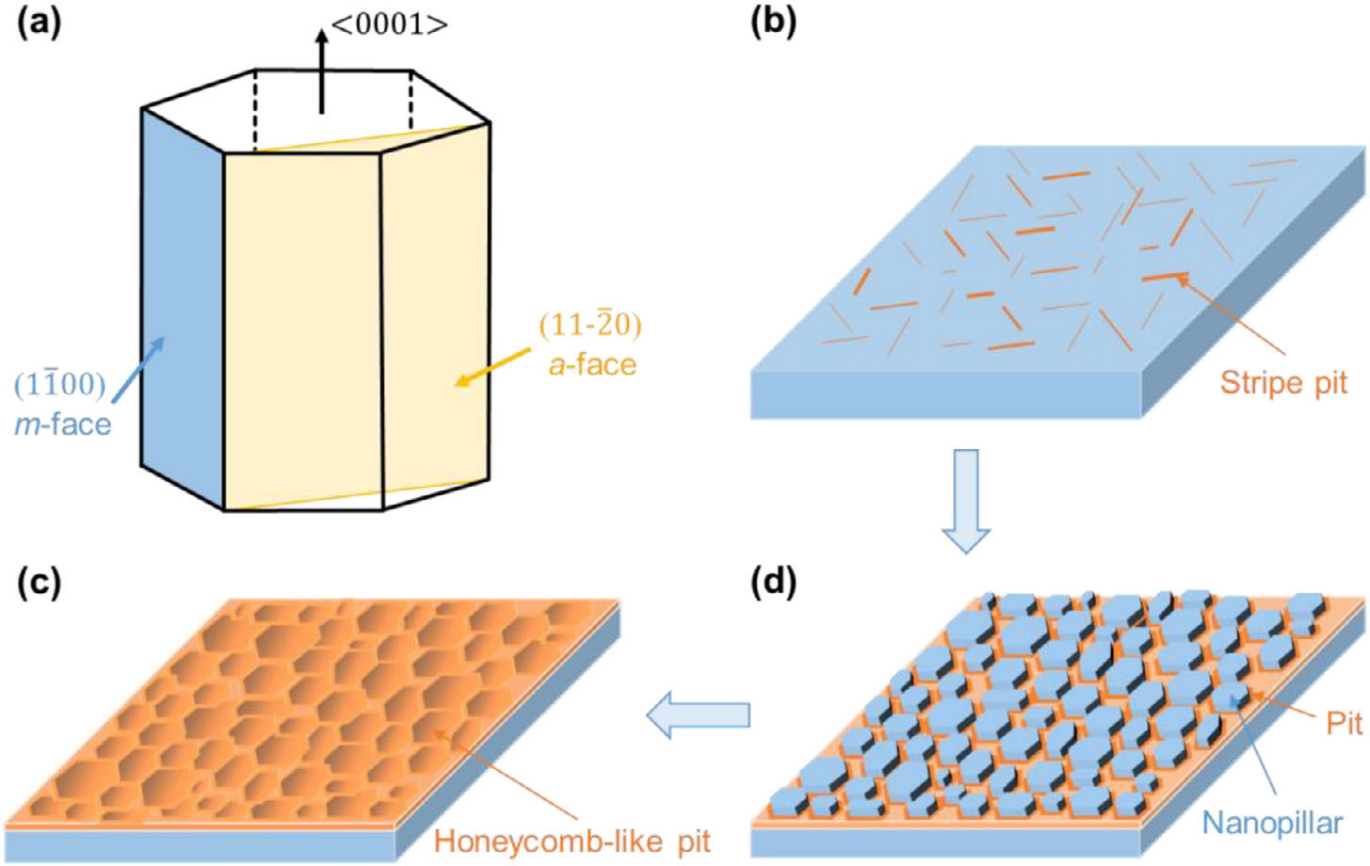
Schematic diagram showing the mechanism of the PEC etching for forming the honeycomb‐like pit on the SiC epilayer. (a) Lattice structure of 4H‐SiC showing (11$\bar{2}$0) *a*‐face (yellow) and (1$\bar{1}$00) *m*‐face (blue). (b–d–c) The process diagram illustrates the formation of a honeycomb‐like pit in the SiC epilayer surface via the PEC etching in the constant current mode.

The formation of the honeycomb‐like microstructures is attributed to the anisotropic etching of SiC by the PEC etching. Figure 2 provides a simplified model to elucidate the formation mechanism of the honeycomb‐like microstructures. The primary crystal planes of 4H‐SiC include the (0001) Si‐face, the (000$\bar{1}$) C‐face, the (11$\bar{2}$0) *a*‐face, and the (1$\bar{1}$00) *m*‐face [36]. Based on atomic density and experimental observations, the anisotropic etching rates follow the order: *m*‐face > *a*‐face > Si‐face [33, 37]. Since the epilayer surface is Si‐face, the PEC etching initiates at multiple nucleation sites at Si‐face and proceeds preferentially along the m‐face direction, forming the stripe‐like etch pits (Figure 2b). Please note that the *m*‐plane possesses three fundamental crystal directions, and thus the etching progresses along these three crystal directions or the opposite sides, which builds the basis for the formation of the hexagonal pits. Nevertheless, the PEC etching also occurs along the *a*‐face direction, which results in the deviation of the pit shape from the perfect hexagons. Then, the continued PEC etching leads to the interconnection of these hexagonal pits, resulting in discrete bamboo‐like nanopillars (Figure 2d). With further etching, the bases of these nanopillars are undercut, causing them to detach and ultimately form the honeycomb‐like microstructures (Figure 2c). The formation process of the honeycomb‐like pits in Figure 2 corresponds to the etched epilayer surface morphologies observed under different conditions, as shown in Figure S5.

**FIGURE 3:**
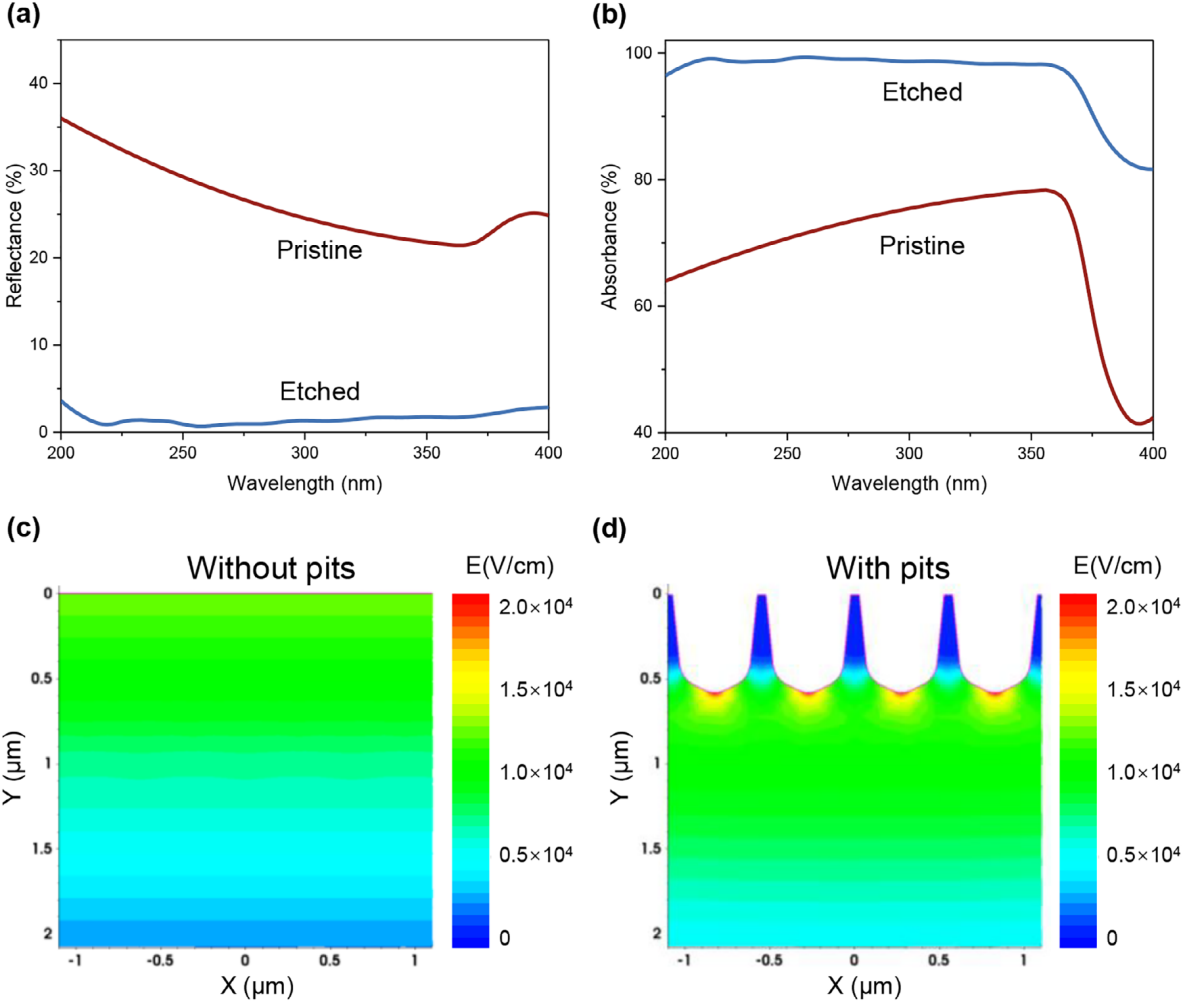
The optical properties of the SiC epitaxial wafer with the honeycomb‐like light‐trapping microstructures. (a) Reflectance and (b) absorbance spectra of the SiC samples before and after the PEC etching at the conditions of 15 mA and 2 h. Local electric field distributions of the SiC ultraviolet photodetectors (c) without and (d) with the honeycomb‐like light‐trapping pits at the bias of 0 V.

To evaluate the light‐trapping ability of the etched SiC epitaxial wafers, reflectance and absorbance spectra were acquired using a UV–vis–NIR spectrophotometer (Figure 3). The pristine sample has the reflectance spectrum consistent with the literature [38], which adheres to the principle of Fresnel's equation. After the PEC etching, a substantial decrease in reflectance was observed in the UV region, with the reflectance remaining below 4% over the 200–400 nm wavelength range. Notably, at the shorter wavelengths, the reflectance at 200 nm was reduced by more than 30%. It can be inferred that the honeycomb‐like light‐trapping microstructures effectively suppress reflection and enhance UV absorption. The honeycomb‐like microstructure demonstrates absorptance greater than 96% within the 200–360 nm spectral range, which plays a crucial role in improving the performance of the SiC‐based ultraviolet photodetectors. The underlying mechanism is that the textured surface facilitates multiple internal reflections and prolongs the optical path length, thereby improving light absorption efficiency. Besides, a simplified device structure model was established based on a honeycomb‐like pit structure and subjected to TCAD simulations. Simulation results reveal that significantly higher electric fields occur at the bottom of the honeycomb‐like pits in the device (Figure 3d) compared to the flat device (Figure 3c). This observation confirms that the honeycomb‐like light‐trapping microstructure can also enhance the local electric field, which is also beneficial for improving the performance of the photodetector [39, 40].

**FIGURE 4:**
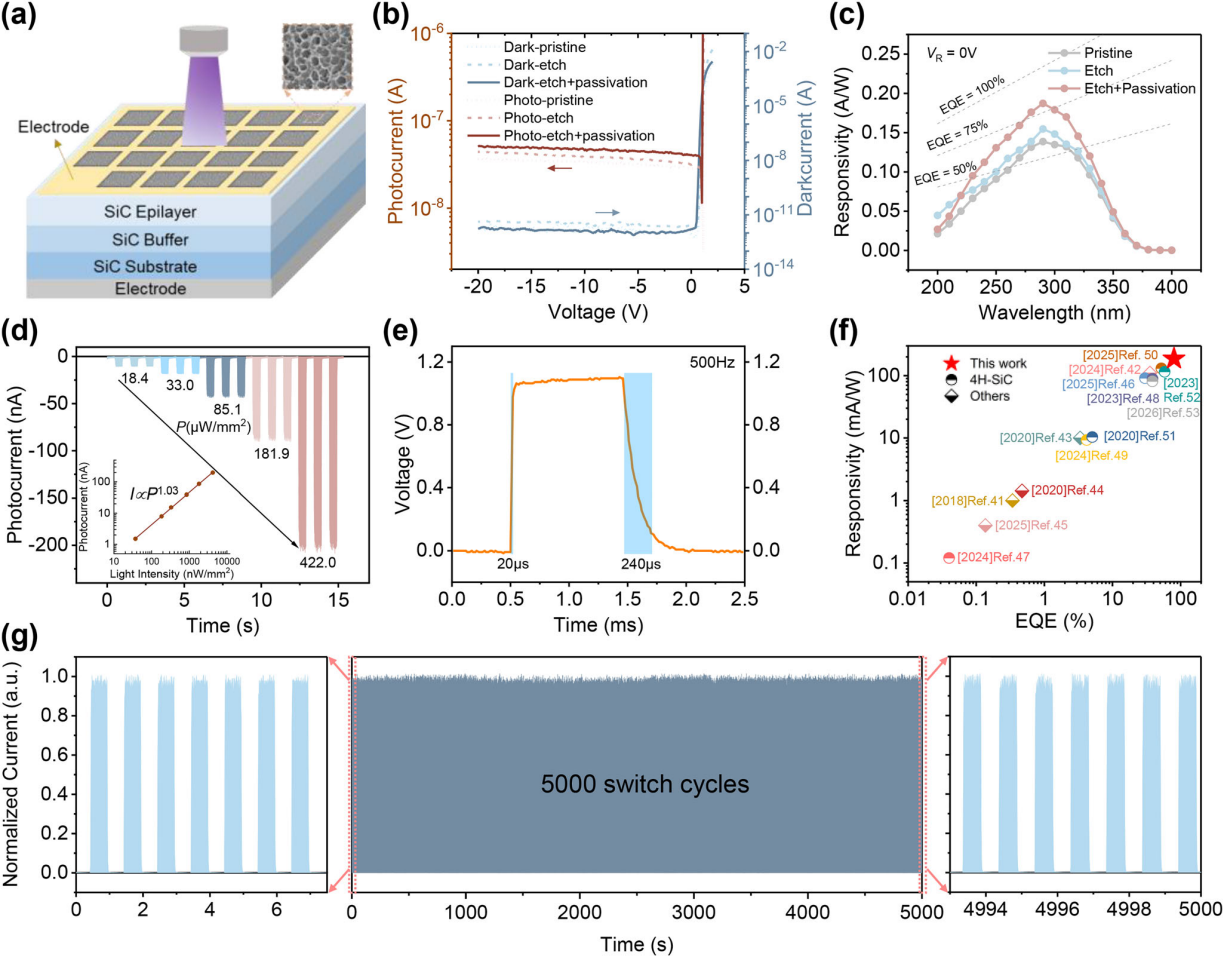
The UV detection performance of the SiC UV PDs with the honeycomb‐like light‐trapping microstructures. (a) Schematic illustration of the vertical Schottky barrier diode (SBD) ultraviolet photodetector with a mesh electrode and the honeycomb‐like light‐trapping microstructure. (b) The dark and the light I–V characteristics of the UV PDs under 261 nm illumination. (c) The responsivity of the UV PDs under the self‐powered operation. (d) *I–t* characteristics and their linear fitting of the optimized SiC UV PDs at different illumination intensities under 300 nm at 0 V. (e) Response time of the UV photodiodes measured using a pulsed laser with a frequency of 500 Hz. (f) Performance comparison of the self‐powered photodetector in this work with those reported in the literatures [41, 42, 43, 44, 45, 46, 47, 48, 49, 50, 51, 52, 53]. (g) Repeated switch cycles of the photodetector under 300 nm UV illumination at 0 V.

We next fabricated and then characterized the performance of the SiC UV photodetectors (PDs) incorporating the honeycomb‐like light‐trapping microstructures. To enable self‐powered UV detection, a vertical Schottky barrier diode (SBD) structure was fabricated, as illustrated in Figure 4a. The front electrode is a mesh design, with the surrounding area etched to form the honeycomb‐like light‐trapping microstructures for enhancing light absorption. To mitigate surface defects introduced by etching, a 20 nm thick Al_2_O_3_ passivation layer was deposited via atomic layer deposition (ALD). Current–voltage measurements under dark and illuminated conditions (261 nm) revealed a significant enhancement in photocurrent and a slight suppression of dark current after the PEC etching and passivation (Figure 4b). The honeycomb‐like light‐trapping microstructures created by etching increase the surface area of SiC, which leads to more dangling bonds. These interface states act as recombination centers for carriers, thereby causing an increase in the dark current. Nevertheless, by depositing an Al_2_O_3_ passivation layer on the SiC surface, the interface state density was effectively reduced via the dangling bond termination, leading to an effective decrease in the device's dark current. Spectral responsivity measurements across 200–400 nm showed substantial improvement in the etched and passivated sample, with a peak responsivity of 187 mA/W at 290 nm, corresponding to an external quantum efficiency of 80% and a specific detectivity of 1.94×10^13^ Jones, as shown in Figure 4c. The relevant calculation formulas are included at the end of the supporting information. The self‐powered *I–t* characteristics of the optimized SiC photodetector at different light intensities are shown in Figure 4d. It is evident that the photocurrent increases with increasing incident light intensity. The logarithmic plot inserted in Figure 4d quantifies the relationship between photocurrent and light intensity, with the fitting result of *I*∝*P* ^1.03^, indicating a strong linear correlation between photocurrent and light intensity. Additionally, the photodetector exhibits a rapid response time under self‐powered conditions, with an upward response time of 20 µs (Figure 4e), suggesting potential applications in low‐power optical communication systems. Moreover, the device performance was compared with other ultraviolet detectors using peak responsivity and quantum efficiency, as shown in Figure 4f. The details of these device characteristics can be found in Table S1. Although the peak wavelengths vary, these comparisons still have comparative value. The results indicate that our device exhibits excellent performance compared to others in the literature. Last, repeated switching cycle tests were conducted to demonstrate the photodetector's stability. As shown in Figure 4g, a switching period of one second was applied to the photodetector. Even after 4000 cycles, the response current exhibited negligible decay. These results indicate that the photodetector demonstrates long‐term stability under ultraviolet illumination and possesses practical functionality.

**FIGURE 5:**
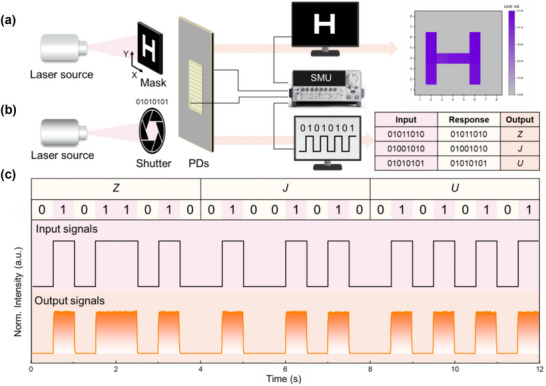
Applications of the self‐powered SiC UV PDs in the single‐device optical communication and imaging system. The schematic diagram of the setups for the (a) imaging applications and (b) optical communication. The output images obtained from a mask with a letter “H,” captured by a single‐pixel imager under 261 nm illumination. (c) The input and output signals are transmitted using the ASCII code of ‘ZJU’ through the optical communication system.

The self‐powered SiC UV PDs with the honeycomb‐like light‐trapping microstructures exhibit excellent detection performance, demonstrating strong potential for various optoelectronic applications. A key factor contributing to their utility is their fast response speed in self‐powered mode, with measured rise and fall times of 20 and 240 µs, respectively. This rapid response is fundamental to the successful implementation of both the imaging and optical communication systems depicted in Figure 5. Figure 5 illustrates the schematic diagrams of a single‐device imaging system and an optical communication setup. In the imaging system (Figure 5a,b), a 261 nm laser emitter and a movable “H”‐shaped mask are able to modulate the photocurrent of the PD, which is decoded into a two‐dimensional image. Similarly, in the optical communication system, the laser output is controlled by a shutter, and the PD's photocurrent is recorded and converted into an ASCII code. Figure 5c demonstrates the successful generation and decoding of a binary data stream containing the information of “ZJU.” The high‐speed optical response and high switching ratio of our PD under self‐powered conditions ensure reliable and accurate optical communication.

## 4 Conclusions

In summary, we have developed a simple and effective photoelectrochemical etching method to fabricate the honeycomb‐like light‐trapping microstructures on the surface of SiC epilayers. This approach enables highly selective etching of the epitaxial layer while preserving the substrate and maintaining the crystalline quality of the SiC material. The formation mechanism underlying the honeycomb‐like microstructure was systematically investigated, revealing its dependence on etching parameters and the anisotropic etching behavior of SiC crystal planes. Optical characterization demonstrates that the introduced microstructures significantly suppress UV reflection and enhance absorption, particularly in the short‐wavelength region. Furthermore, a self‐powered Schottky junction UV photodetector incorporating the optimized light‐trapping microstructure was fabricated and evaluated. This device exhibits a remarkable peak responsivity of 187 mA/W at 290 nm, along with an external quantum efficiency of 80%, outperforming most previously reported SiC‐based photodetectors. The low‐cost, scalable, and selective nature of this PEC etching strategy offers a promising route for enhancing the performance of the SiC optoelectronic devices, especially in visible‐blind UV detection applications.

## Conflicts of Interest

The authors declare no conflicts of interest.

## Supporting information




**Supporting File**: advs74352‐sup‐0001‐SuppMat.docx

## Data Availability

The data that support the findings of this study are available from the corresponding author upon reasonable request.
