# Supplementary material for: Ultraviolet Photodetectors Based on 4H‐SiC With Honeycomb‐Like Light‐Trapping Structures
Source: Adv Sci (Weinh). 2026 Feb 11;13(22):e74352. doi: 10.1002/advs.74352 (PMC13088326; doi:10.1002/advs.74352)
Supplement: Supplementary file 1 — Supporting File: advs74352‐sup‐0001‐SuppMat.docx [file ADVS-13-e74352-s001.docx]

SUPPORTING INFORMATION

Ultraviolet Photodetectors Based on 4H-SiC with Honeycomb-like Light-Trapping Structures

Huifan Xiong^1,2^, Xiliang Luo^1,2^, Qunsi Yang^2*^, Yibo Hu^3^, Jieshi Chen, Lihui Song^1,2*^, Deren Yang^1,2^, Xiaodong Pi^1,2*^

^1^ State Key Laboratory of Silicon and Advanced Semiconductor Materials & School of Materials Science and Engineering, Zhejiang University, Hangzhou, China.

^2^ Key Laboratory of Power Semiconductor Materials and Devices of Zhejiang Province & Institute of Advanced Semiconductors, ZJU-Hangzhou Global Scientific and Technological Innovation Center, Zhejiang University, Hangzhou, China.

^3^ School of Materials Science and Engineering, Shanghai University of Engineering Science, Shanghai, China.

^*^Corresponding emails:[songlihui@zju.edu.cn](mailto:songlihui@zju.edu.cn), [qunsi@zju.edu.cn](mailto:qunsi@zju.edu.cn) and [xdpi@zju.edu.cn](mailto:xdpi@zju.edu.cn).

The 4H-SiC epitaxial wafer was characterized using Raman, XRD, and HRTEM. It was observed in the Raman spectrum that the transverse optical (FTO) mode at 777.4 cm^-1^ exhibits maximum intensity for a reduced wave vector x = 0.5, thereby confirming that the sample is 4H-SiC^[S1, 2]^. A sharp peak at 35.2° was revealed by the XRD analysis, corresponding to the (0004) crystallographic plane of 4H-SiC^[S3]^. Additionally, a FWHM of 78.8 arc seconds was exhibited by the rocking curve, indicating excellent single-crystal quality. HRTEM imaging confirmed this as well, with periodic lattice fringes being displayed. These results demonstrate the superior crystalline quality of the 4H-SiC sample, providing a fundamental basis for the high performance of the fabricated devices.


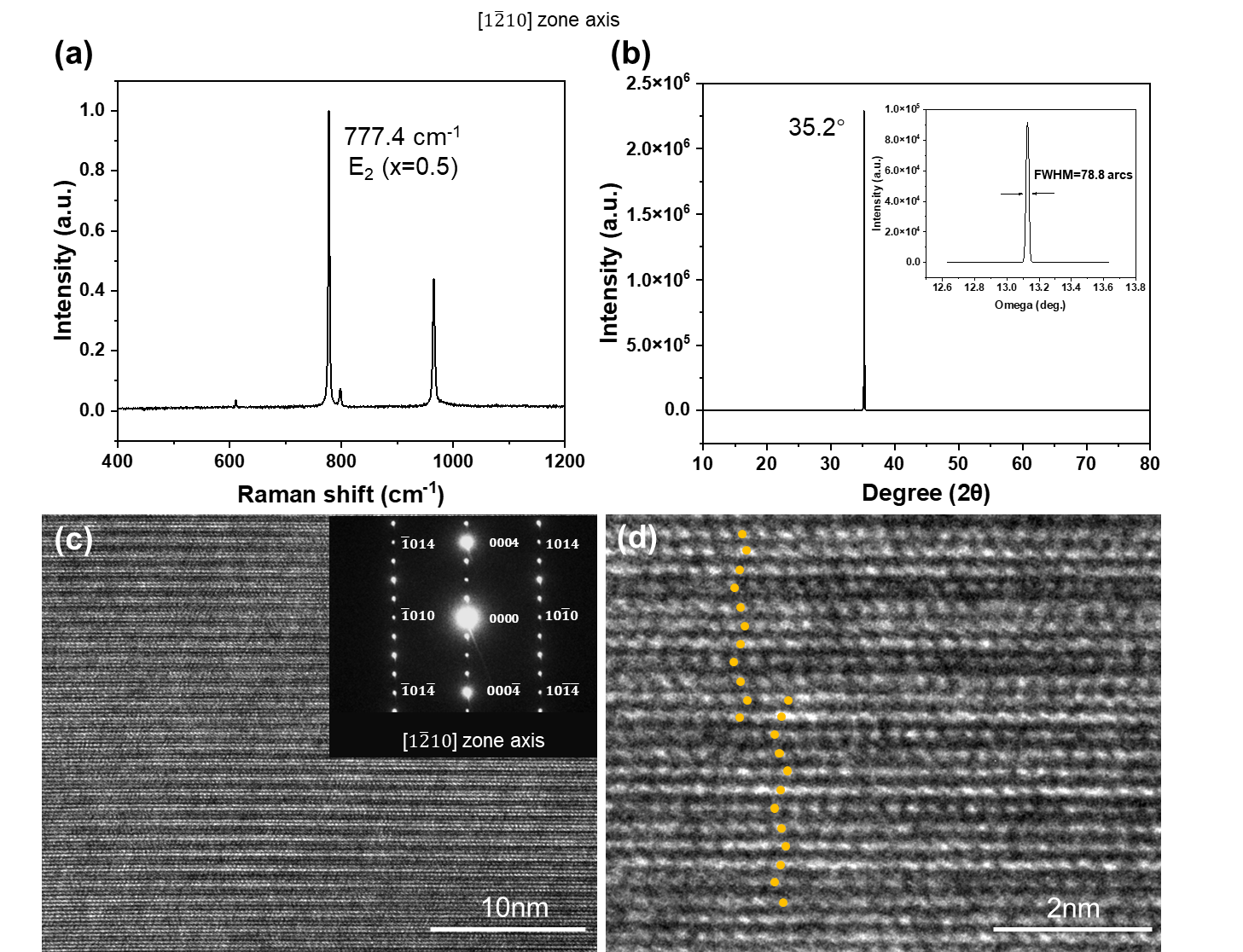


**Figure S1. (a)** Raman spectrum and **(b)** XRD results of the 4H-SiC sample. The inset image presents the rocking curve measurement, yielding a FWHM of 78.8 arc seconds. **(c)** HRTEM image and **(d)** local magnified view of 4H-SiC along the [1$\bar{2}$10] direction, with the inset showing the electron-diffraction spots.


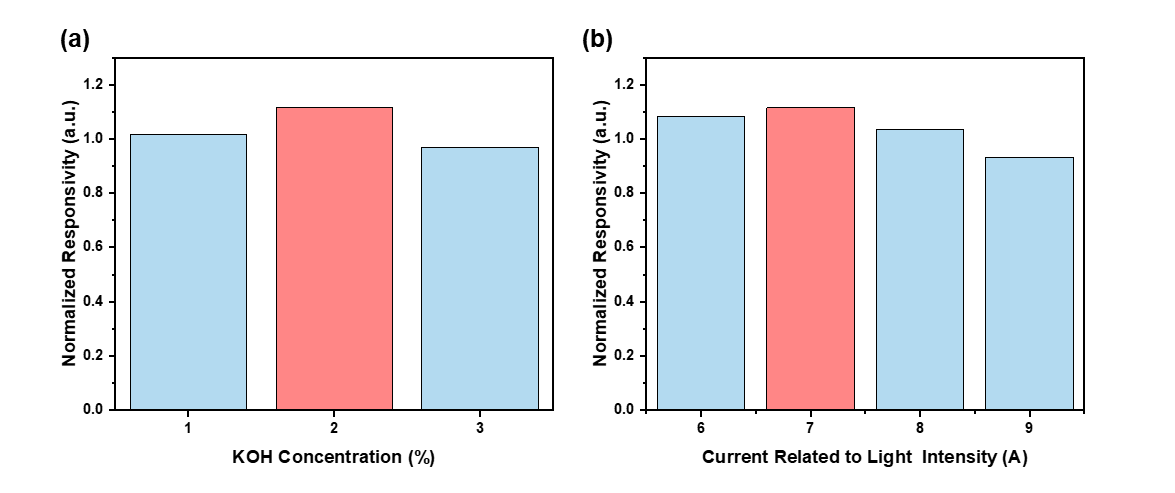


**Figure S2. Etching parameter optimization process.** Comparative results of peak responsivity at 290nm after etching under different **(a)** KOH concentrations (1%, 2%, 3%) and **(b)** mercury lamp light intensities, where intensity was varied by the operating currents (6A, 7A, 8A, 9A).


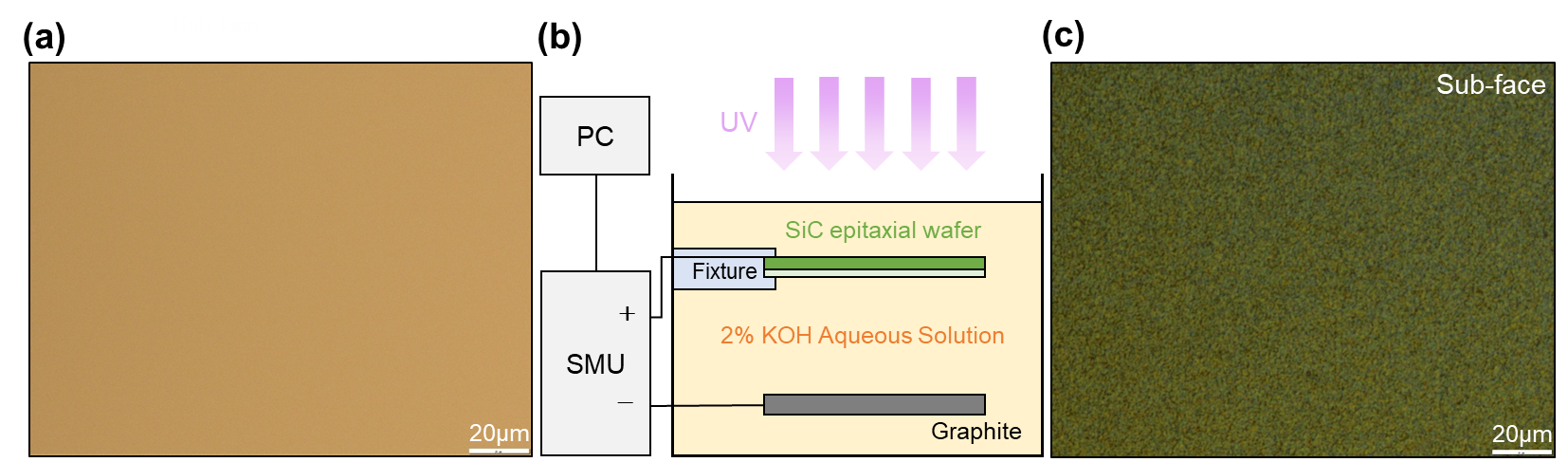


**Figure S3. (a)** DIC dark field images of substrate surface of an etched sample. **(b)** Schematic illustration of the PEC etching set-up for the control experiment, where the SiC epitaxial wafer is fully immersed in the PEC etching solution. **(c)** dark field image of the substrate surface in the control experiment measured via DIC microscopy.


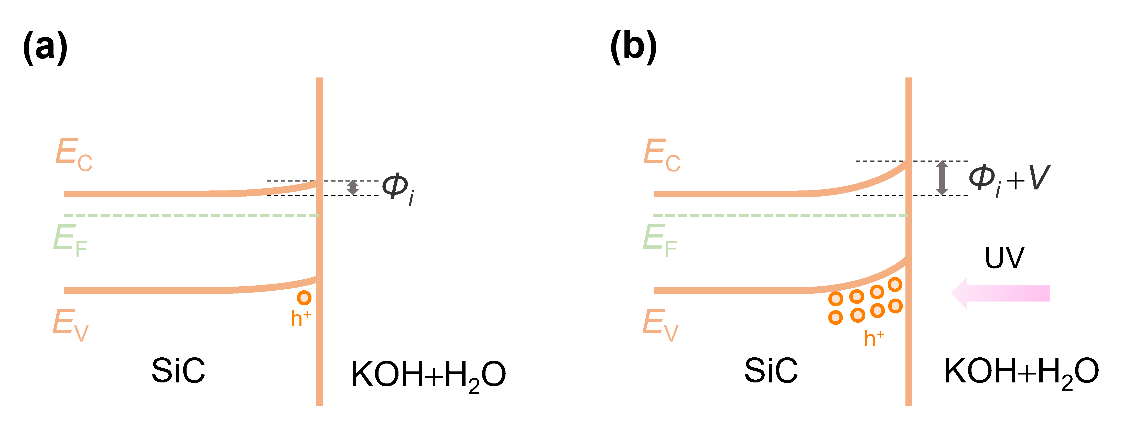


**Figure S4.** **Energy band diagram showing the mechanism of the PEC etching on the SiC surface.** **(a)** Energy band diagram of the SiC without applied bias and UV illumination. **(b)** Energy band diagram of the SiC under bias voltage and UV illumination, in which case holes accumulate at the SiC surface.


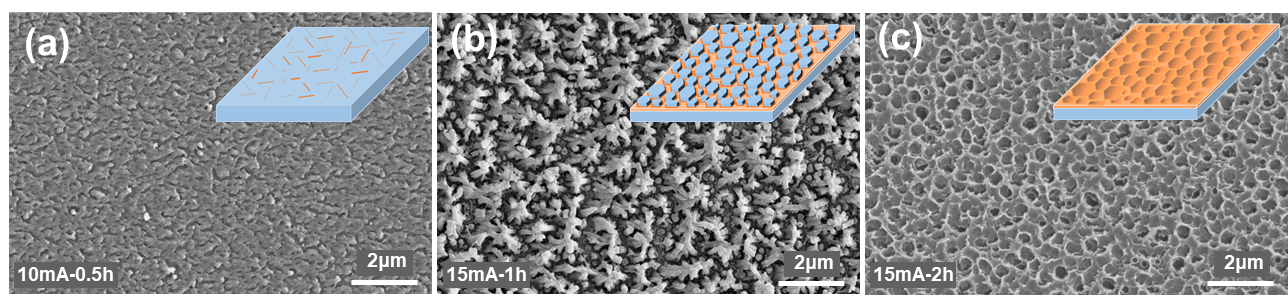


**Figure S5. Images of pit morphology formed by the PEC etching under different conditions (a) 10 mA for 0.5 h, (b) 15 mA for 1 h, (c) 15mA for 2h.**


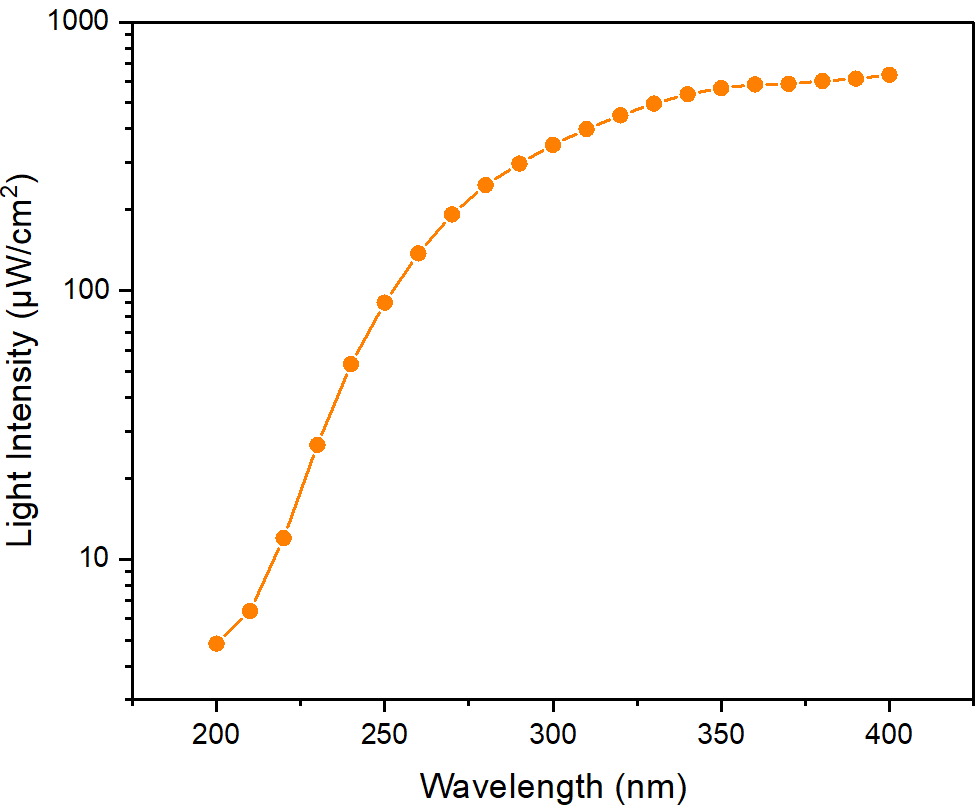


**Figure S6. The curve of light intensity versus wavelength measured during the tests of spectral response and quantum efficiency for the photodetectors.**

According to the thermionic emission theory, the current transmission formula for a Schottky contact can be expressed as:

$$I=AA^{*}T^{2}\exp\left( -\frac{q\Phi_{b}}{kT} \right)[\exp\left( \frac{qV}{nkT} \right)-1]$$

where A is the Schottky electrode area, A* is the effective Richardson constant, *Φ_b_* is the Schottky barrier height, and *n* is ideality factor.

In a semi-logarithmic coordinate system, the intermediate linear region is fitted to obtain a linear equation. From the intercept and the slope of this fitted line, the following relationships can be derived:

$$\Phi_{b}=\frac{kT}{qlne}ln|\frac{AA^{*}T^{2}}{Intercept}|$$

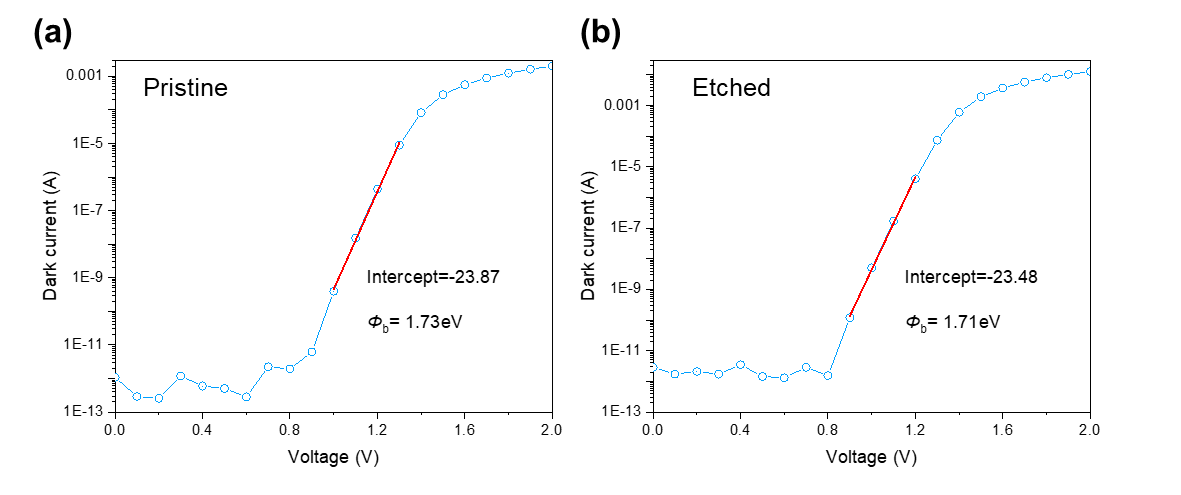


**Figure S7. Forward bias I–V curves of the (a) pristine and (b) etched SiC photodetectors on semi-logarithmic coordinates.**

**Table S1. Device characteristics of the self-powered photodetector in this work compared with those reported in the literatures.** Note: The numbered references in the normal font in the table correspond to the references in the manuscript, while the numbers in the superscript brackets refer to the reference numbers in the supporting information.

| **Sample** | **λ (nm)** | **R (mA/W)** | **EQE (%)** | **D* (Jones)** | **τ_R_(ms)** | **Ref.** |
| --- | --- | --- | --- | --- | --- | --- |
| 4H-SiC | 290 | 187 | 80 | 1.94×10^13^ | 0.02 | This work |
| ZnO | 360 | 1 | 0.34 | ~2.5×10^10^ | 3900 | 41^[S4]^ |
| Cs_2_AgBiBr_6_/ SnO_2_ | 350 | 110 | 40 | 2.1×10^10^ | 2 | 42^[S5]^ |
| Cs_2_AgBiCl_6_ | 370 | 9.68 | 3.35 | 1.11×10^12^ | − | 43^[S6]^ |
| ZnO | 375 | 1.42 | 0.47 | 1.07×10^11^ | 1250 | 44^[S7]^ |
| CuO/*α*-Ga_2_O_3_ | 254 | 0.4 | 0.136 | 9.7×10^7^ | − | 45^[S8]^ |
| 4H-SiC/ZnS | 375 | 91 | 30.24 | 1.69×10^11^ | − | 46^[S9]^ |
| 4H-SiC/Si | 365 | 0.12 | 0.04 | 9.2×10^6^ | 37.6 | 47^[S10]^ |
| 4H-SiC/  graphene | 300 | 89 | 38.7 | 2.9×10^12^ | 0.009 | 48^[S11]^ |
| 4H-SiC/  graphene | 275 | 9.27 | 4.18 | 1.21×10^8^ | − | 49^[S12]^ |
| 4H-SiC/CuI | 310 | 130.7 | 52.28 | 5.73×10^12^ | 7.3 | 50^[S13]^ |
| 4H-SiC/  *β*- Ga_2_O_3_ | 254 | 10.35 | 5.05 | 8.8×10^9^ | 11 | 51^[S14]^ |
| 4H-SiC/  ZnGa_2_O_4_ | 244 | 115 | 58.4 | ~9.6×10^10^ | 18.36 | 52^[S15]^ |
| 4H-SiC | 260 | 81 | 38.63 | 2.9×10^8^ | 47.12 | 53^[S16]^ |

The photodetector performance is quantitatively evaluated through responsivity (*R_λ_*) , external quantum efficiency (*EQE*) and specific detectivity (*D^*^*), which are calculated as follows^[S6, 12, 17-20]^:

$$R=\frac{I_{ph}-I_{dark}}{PA}$$

$$EQE=\frac{hc}{e}R$$

$$D^{*}=\frac{R}{{(2e\frac{I_{dark}}{A})}^{\frac{1}{2}}}$$

where *I_ph_*, *I_dark_*, *P*, *A*, and *e* represent photo current, dark saturation current, light intensity, effective area, and the elemental charge of an electron, respectively.

**Reference**

S1. Z. C. Feng, D. Zhao, L. Wan, et al., "Angle-Dependent Raman Scattering Studies on Anisotropic Properties of Crystalline Hexagonal 4H-SiC," *Materials (Basel)* **15**, no.24 (2022).

S2. W. Geng, Q. Shao, Y. Pei, et al., "Slicing of 4H-SiC Wafers Combining Ultrafast Laser Irradiation and Bandgap-Selective Photo-Electrochemical Exfoliation," *Advanced Materials Interfaces* **10**, no.21 (2023): 2300200.

S3. G. Kumar, J.-W. Chen, H.-H. Ma, X.-F. Huang M. Huang, "Facet-dependent electrical conductivity properties of 4H-SiC wafer," *Journal of Materials Chemistry C* **10**, no.28 (2022).

S4. Y. Ning, Z. Zhang, F. Teng X. Fang, "Novel Transparent and Self-Powered UV Photodetector Based on Crossed ZnO Nanofiber Array Homojunction," *Small* **14**, no.13 (2018): 1703754.

S5. C. Wu, B. Du, W. Luo, et al., "Highly Efficient and Stable Self-Powered Ultraviolet and Deep-Blue Photodetector Based on Cs_2_AgBiBr_6_/SnO_2_ Heterojunction," *Advanced Optical Materials* **6**, no.22 (2018): 1800811.

S6. M. Wang, P. Zeng, Z. Wang M. Liu, "Vapor-Deposited Cs_2_AgBiCl_6_ Double Perovskite Films toward Highly Selective and Stable Ultraviolet Photodetector," *Advanced Science* **7**, no.11 (2020): 1903662.

S7. Y. Zhang, M. Peng, Y. Liu, et al., "Flexible Self-Powered Real-Time Ultraviolet Photodetector by Coupling Triboelectric and Photoelectric Effects," *ACS Applied Materials & Interfaces* **12**, no.17 (2020): 19384-19392.

S8. J. Zhang, X. Guo, W. Bai, et al., "Self-powered wide bandgap UV detector based on CuO/α-Ga2O3 heterostructure," *Applied Physics A* **131**, no.1 (2024): 50.

S9. B. Zou, Y. Yang, H. Wang, et al., "High-Responsivity Self-Powered Photoelectrochemical UV Photodetector Based on Integrated Self-Supporting SiC/ZnS Heterojunction Nanowire Arrays," *Small* **21**, no.5 (2025): 2406308.

S10. L. Li, G. Wei, P. Zhu, et al., "Self-Powered Solar-Blind Photodetector With Low Dark Current and Ultrahigh On/Off Ratio Based on Vertically Aligned Si/SiC Nanowire Arrays p-n Heterojunction," *IEEE Trans Electron Devices* **71**, no.7 (2024): 4173-4179.

S11. A. K. Jehad, M. Fidan, Ö. Ünverdi C. Çelebi, "CVD graphene/SiC UV photodetector with enhanced spectral responsivity and response speed," *Sensors and Actuators A: Physical* **355**, no. (2023): 114309.

S12. L. Li, G. Wei, P. Zhu, et al., "Self-powered graphene/4H-SiC nanowire array-based ultraviolet photodetectors with fast response time and low dark current for promising wireless ultraviolet communication," *Applied Materials Today* **37**, no. (2024): 102114.

S13. L. Yang, X. Wang, Y. Shan, et al., "Self-Driven Ultraviolet Light and Imaging Detectors Composed of CuI/4H-SiC Heterostructures With an Ultrahigh Photovoltage Responsivity," *IEEE Trans Electron Devices* **72**, no.8 (2025): 4170-4176.

S14. J. Yu, L. Dong, B. Peng, et al., "Self-powered photodetectors based on β-Ga2O3/4H–SiC heterojunction with ultrahigh current on/off ratio and fast response," *Journal of Alloys and Compounds* **821**, no. (2020): 153532.

S15. D. Han, Y. Yang, L. Meng, et al., "High-performance self-powered solar-blind ultraviolet photodetector based on a 4H-SiC/ZnGa2O4 heterojunction and its application in optical communication," *Appl Phys Lett* **123**, no.20 (2023): 201104.

S16. A. M. Dülcel, M. Gözek, Ö. Ünverdi C. Çelebi, "Comparison of the photoresponse characteristics for 4H–SiC Schottky barrier UV photodetector with graphene and Ni/Cr electrode," *Opt. Mater.* **173**, no. (2026): 117854.

S17. I. B. Khadka, N. R. Alluri, M. M. Alsardia, et al., "Ultra-low-power photodetector based on a high-photoresponse, plasmonic-effect-induced gateless quasi-freestanding graphene device," *Applied Surface Science* **610**, no.1 (2023): 155275.

S18. X. Wang, H. Li, Q. Wei, et al., "Gradient Passivation of Oxygen Vacancy Boosts Ga2O3 Solar-Blind UV Detector," *Advanced Materials Technologies*  no.e01970 (2025): 8.

S19. X. Zhou, L. Zhang, Y. Huang, et al., "Enhanced Responsivity of CsCu2I3 Based UV Detector with CuI Buffer-Layer Grown by Vacuum Thermal Evaporation," *Advanced Optical Materials* **9**, no.20 (2021): 2100889.

S20. M. Hoang Tran, J.-S. Bae J. Hur, "Self-powered, transparent, flexible, and solar-blind deep-UV detector based on surface-modified TiO2 nanoparticles," *Applied Surface Science* **604**, no. (2022): 154528.
